# Vision-Based Jigsaw Puzzle Solving with a Robotic Arm

**DOI:** 10.3390/s23156913

**Published:** 2023-08-03

**Authors:** Chang-Hsian Ma, Chien-Liang Lu, Huang-Chia Shih

**Affiliations:** Department of Electrical Engineering, Yuan Ze University, Taoyuan 32003, Taiwan

**Keywords:** edge similarity, Hausdorff distance, patch reconstruction, puzzle solving, robotic arm, template matching

## Abstract

This study proposed two algorithms for reconstructing jigsaw puzzles by using a color compatibility feature. Two realistic application cases were examined: one involved using the original image, while the other did not. We also calculated the transformation matrix to obtain the real positions of each puzzle piece and transmitted the positional information to the robotic arm, which then put each puzzle piece in its correct position. The algorithms were tested on 35-piece and 70-piece puzzles, achieving an average success rate of 87.1%. Compared with the human visual system, the proposed methods demonstrated enhanced accuracy when handling more complex textural images.

## 1. Introduction

Jigsaw puzzles are commonly enjoyed by children and adults. The pictures used in jigsaw puzzles mostly display natural scenes and buildings. A jigsaw puzzle usually comprises puzzle pieces with a rectangular profile; its content is often informative, and its color and outline contrasts are sharp. Furthermore, each corner piece of a jigsaw puzzle has a recognizable component of the picture or design of the puzzle. Therefore, humans can piece together specific parts of a puzzle and subsequently put all the pieces together. When the original image is available, a player identifies specifics of the region of a set of puzzle pieces and compares them with the original image. Subsequently, the player classifies the puzzle pieces into various groups via feature similarity. They check the puzzle pieces’ colors, shapes, or patterns to determine whether two puzzle pieces are adjacent, and this process is repeated until the puzzle is completed. However, in cases where the original image is unavailable, solving the puzzle reconstruction becomes more challenging. The only available clues are image-based characteristics, such as color, edges, and specific templates. With numerous possible combinations to consider, it significantly increases the difficulty of puzzle solving. The trial-and-error approach is often employed, but its main drawback is inefficiency.

When a computer-vision-based solution is applied, directly comparing the colors or patterns on the edges of a jigsaw puzzle for continuity is a feasible approach. However, calculating the level of similarity between two puzzle pieces is a highly complex and time-consuming task. Therefore, when the original image is available, we can focus on the features of the puzzle pieces and compare them with those of the image. The goal is to determine the relationship between the puzzle and the image’s features. To solve this problem, the present study applied the scale-invariant feature transform (SIFT) [1] to search for features in an image and then used a random sample consensus (RANSAC) [2] algorithm to determine the optimal combination for whole pieces.

Solving jigsaw puzzle problems is a challenging but extensively researched task in computer vision research. Researchers proposed image processing and pattern recognition methods for solving jigsaw problems [3]. Those solutions consider the shape of each puzzle piece and the similarity between adjacent pieces. In general, the methods for solving jigsaw puzzle problems can be classified into two types. The first type involves the use of the original image, whereas the second type does not involve the use of this information. Li et al. [4] applied the SIFT algorithm to extract the features of each puzzle piece and those of the original image of a jigsaw puzzle. The features of each piece were matched to the regions of the original image that exhibited the most similarity with them. Furthermore, some researchers examined the relationship of puzzle pieces with specific shapes. The robust template-matching algorithm [5] is suitable for solving the aforementioned problem.

Without using original images, Demaine and Demaine [6] indicated that solving jigsaw puzzles is an NP-hard problem. For problems involving fossil reconstruction [7], archaeological finds [8], torn images, and document reconstruction [9,10,11], Willis and Cooper [12] proposed two-dimensional and three-dimensional methods. Leitão and Stolfi [13] formulated a tile reconstruction algorithm with dynamic programming. Andalá et al. [14] applied quadratic programming and gradient ascent methods to solve puzzles. These studies highlighted that designing an efficient algorithm is crucial. When the puzzle pieces of a jigsaw puzzle are rectangular, all pieces share the same feature with respect to their shape; therefore, these pieces can only be matched by calculating the levels of similarity between the edges of puzzle pieces [15,16,17,18]. In this situation, the aforementioned algorithm would continually check the correctness of a puzzle combination, gradually increasing the size of the puzzle. In addition to the method of feature extraction, the algorithm for obtaining a puzzle combination is crucial. Moussa et al. [19] used a greedy algorithm to identify an optimal puzzle combination. Sholomon et al. [20] used an iterative genetic algorithm to calculate averaged results. Recently, a few solutions for the puzzle reassembly problem were proposed by using deep learning-based [21] and reinforcement-learning-based [22] algorithms. In addition, Bridger et al. [23] presented an approach that involves filling in the eroded boundaries between puzzle pieces by using a generative adversarial network (GAN). Similarly, the puzzle reassembly was formulated as a combinatorial optimization problem and then solved using a genetic algorithm [24].

**Figure 1:**
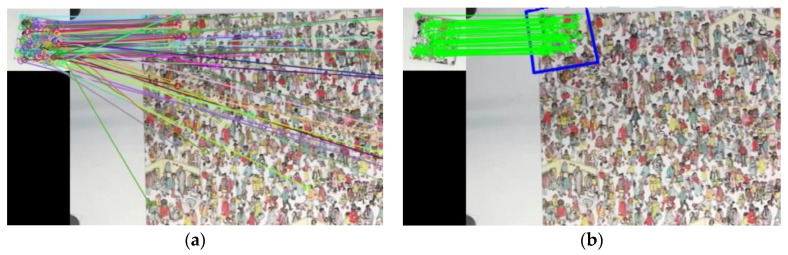
Example of (**a**) SIFT matching pairs and (**b**) filtered pairs using RANSAC, where the blue box denotes the mapped location of the patch with rotation angle in original image.

The present study proposed two algorithms for solving jigsaw puzzle problems. The first algorithm was applied when the original image was available, and SIFT and RANSAC were used to reconstruct a puzzle efficiently. An example of pixel matching and filtering is shown in Figure 1. The second algorithm was applied when the original image was unavailable, and a greedy-based algorithm with a repair mechanism was used to solve the problems presented in this scenario. We also used our algorithms with a robotic arm to demonstrate that our solutions can be applied in a practical setting.

The main contributions of this study are as follows:

(1) A proposed algorithm that is fast and accurate for solving puzzle reconstruction when the original image is available.
(2) The presentation of a lightweight and systematic algorithm for solving puzzle reconstruction without relying on the availability of the original image.
(3) Improved accuracy was observed when dealing with more complex textural images.
(4) The algorithm maintained a linear complexity, regardless of the complexity of the test image.

## 2. The Proposed Algorithms

### 2.1. The Algorithm with the Original Image

SIFT is a widely used algorithm in computer vision research. The SIFT feature descriptor is invariant to rotation, scaling, and illumination, and it is often used to identify the features in target images. The speeded up robust features (SURF) [25] and oriented FAST and rotated BRIEF (ORB) algorithms [26] are similar to SIFT. SURF has an advantage over SIFT in computational speed, but SIFT is more accurate. ORB is faster than SURF, but it does not exhibit scale invariance and is more sensitive to the noise in an image. In the present study, we applied SIFT because of its higher accuracy relative to ORB.

**Figure 2:**
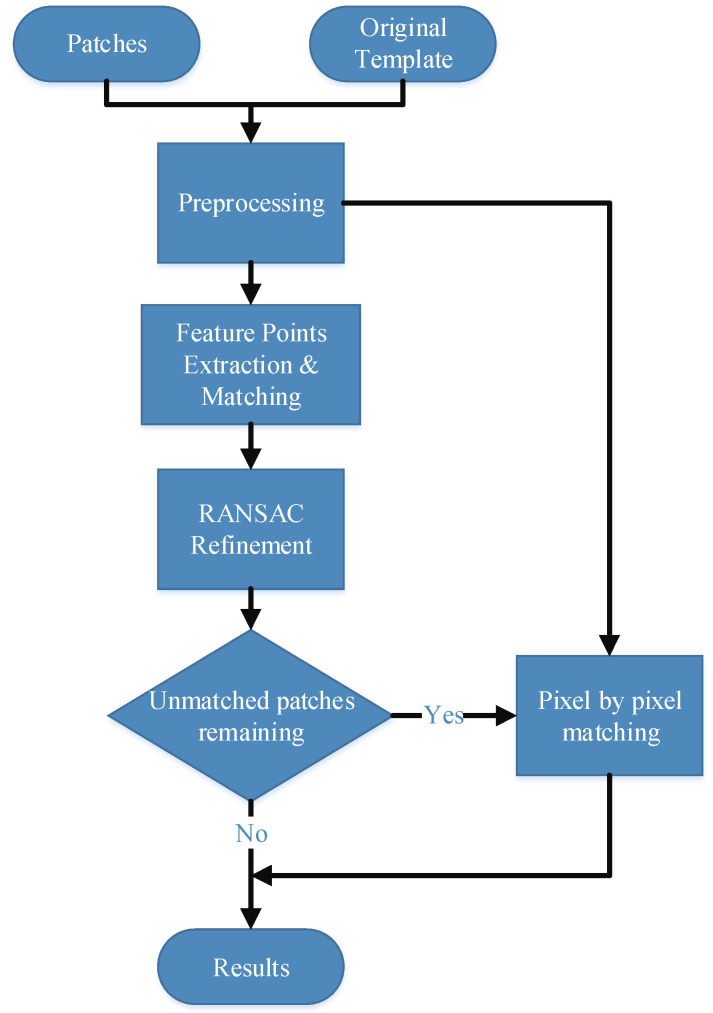
Flowchart of the proposed method with an original image.

The flowchart of the algorithm is shown in Figure 2, which can be divided into three steps, namely, the extraction of feature points, the determination of the directions of feature point gradients, and the building of a SIFT descriptor.

#### 2.1.1. Feature Point Extraction

Each fragmented puzzle piece can be randomly placed at different angles, making rotation invariance necessary. Additionally, scaling may vary slightly due to the distance from which the images are captured, albeit not significantly. However, to maintain the adaptability of the system, it is still essential to have a feature point extraction method that is both scaling and rotation invariant. As a result, we built the difference-of-Gaussian (DoG) pyramid and used the differences between Gaussian blur images at different scales to identify feature points. Through this method, we could ensure that our feature points were scale and rotation invariant. We initially used two different scale images to build a pyramid layer. The first layer was double the size of the original image, the second layer was the original image, and the third and fourth layers were the original image reduced by one and two times, respectively. The scaled Gaussian function is defined as follows:

(1) Gx,y,σ=12πσ2e−x2+y22σ2

A Gaussian blur image and a DoG image are defined as follows:

(2) $$D\left(x,y,\sigma \right)=\left[G\left(x,y,p\sigma \right)-G\left(x,y,\sigma \right)\right]*\;I\left(x,y\right)$$

(3) $$L\left(x,y,\sigma \right)=G\left(x,y,\sigma \right)*I\left(x,y\right)$$

Notably, p is a constant and is usually set to a value of 2. σ is a parameter for Gaussian smoothing. A larger value of σ will result in a broader range being considered, but the proportion of the outermost pixels in the calculation will decrease relative to the inner pixels.

#### 2.1.2. Determining the Direction of Feature Point Gradients

To identify the feature points in a DoG pyramid, each sampling point must be compared with all of its adjacent points. Specifically, a sampling point must be compared with the eight pixels adjacent to it in the middle layer and the nine pixel points in the upper and lower layers. That is, each sampling point must be compared with the 26 points adjacent to it. If a sampling point’s grayscale value is greater than those of these 26 points, it is regarded as a feature point.

#### 2.1.3. Building a SIFT Descriptor

For each feature point, the gradient used to describe the direction of the feature point was calculated. After the feature point direction was obtained, it could be rotated to the main gradient direction for matching. The gradient direction was determined by generating a gradient direction histogram with 16 × 16 pixels around the feature point. The gradient direction *θ* and intensity m of the pixel position (*x*, *y*) in an image are calculated as follows:

(4) $$\theta \left(x,y\right)=\tan^{-1}\left(\frac{L\left(x,y+1\right)-L\left(x,y-1\right)}{L\left(x+1,y\right)-L\left(x-1,y\right)}\right)$$

(5) $$m\left(x,y\right)=\sqrt{(L\left(x+1,y\right)-L\left(x-1,y\right){)}^{2}+(L\left(x,y+1\right)-\left(x,y-1\right){)}^{2}}$$

#### 2.1.4. RANSAC Algorithm

Because a puzzle may exhibit numerous similar local patterns, the feature points detected through SIFT may also generate incorrect matching pairs. However, without the correct matching pairs, the next step puzzle matching matrix will be calculated incorrectly. Therefore, the present study applied the RANSAC [2] algorithm to exclude incorrect matching pairs. The RANSAC algorithm was proposed by Fischler and Bolles, who applied random sampling techniques to identify the parameters of a model from a set of observed data. In the present study, three pairs of feature point matching pairs between origin images and target images were randomly sampled to calculate the transformation matrix. Subsequently, we calculated the distance between each matching pair after performing a transformation procedure. If the distance between matching pairs was less than 3, the pairs were regarded as an inlier group. Subsequently, we randomly selected another three matching pairs and repeated the aforementioned step. After this iterative process was completed, we obtained the transform matrix that contained the highest number of inlier matching pairs.

We set *n* as the number of feature points, $S=\left\{s_{0},s_{1},\ldots ,s_{n}\right\}$ as the feature points in a puzzle image, and $D=\left\{d_{0},d_{1},\ldots ,d_{m}\right\}$ as the distance in a matching set with *m* pairs obtained from SIFT. *k* is the number of iterations used in the RANSAC method; it is employed to match feature point pairs’ relative positional relationships. Increasing *k* can better ensure the removal of mismatched feature point pairs, but it also increases the computational complexity. The main steps of RANSAC performed in the presented study were as follows:

Step 1. Three matching pairs in *S* were randomly sampled, and the transform matrix was calculated using a selected pair.
Step 2. The feature points in a target image were transformed, and a newer $D^{\prime }=\left\{d_{0}^{\prime },d_{1}^{\prime },{\ldots ,d}_{m}^{\prime }\right\}$ was obtained.
Step 3. The distance in $D^{\prime }$ was calculated, and a check was performed to determine whether this distance was less than the threshold $d_{t}^{\prime }$.
Step 4. Steps 1–3 were repeated *k* times, and the maximum inlier pairs were selected as a result.

#### 2.1.5. Transformation Matrix

Assume that *M* denotes a 2-by-3 transformation matrix. It can be obtained using three sets of feature point pairs, which can determine the orientation and, subsequently, the rotation angle. Since the puzzle itself was placed flat on the table and has minimal deformation compared with the camera’s perspective, an affine-based transformation method could be used to achieve this. The set of points $\tilde{S}$ denotes three randomly selected feature points from the puzzle pieces, denoted as $\tilde{S}$ = {*s*_1_,*s*_2_,*s*_3_}. The set of points $\tilde{D}$ represents the set of three target feature points that $\tilde{S}$ is matched to, which is denoted as $\tilde{D}$ = {*d*_1_,*d*_2_,*d*_3_}. Each point in the set represents a coordinate.

(6) $$\left[\begin{array}{c} x_{i}^{\prime }\\ y_{i}^{\prime } \end{array}\right]=M\cdot \left[\begin{array}{c} x_{i}\\ y_{i}\\ 1 \end{array}\right]$$

(7) $$d_{i}=\left(x_{i}^{\prime },y_{i}^{\prime }\right),{\;\;s}_{i}=\left(x_{i},y_{i}\right),\;i=0,1,2$$

Next, we applied the slope and angle formulas to calculate the angle *θ* at which the puzzle fragment is rotated onto the original image. Then, from the contour feature, we could obtain the coordinates of the center point of the puzzle using the following equation:

(8) $$\left(x_{m}^{\prime },y_{m}^{\prime }\right)=(M_{11}x_{m}+M_{12}y_{m}+M_{13},M_{21}x_{m}+M_{22}y_{m}+M_{23})$$

### 2.2. The Algorithm without the Original Image

**Figure 3:**
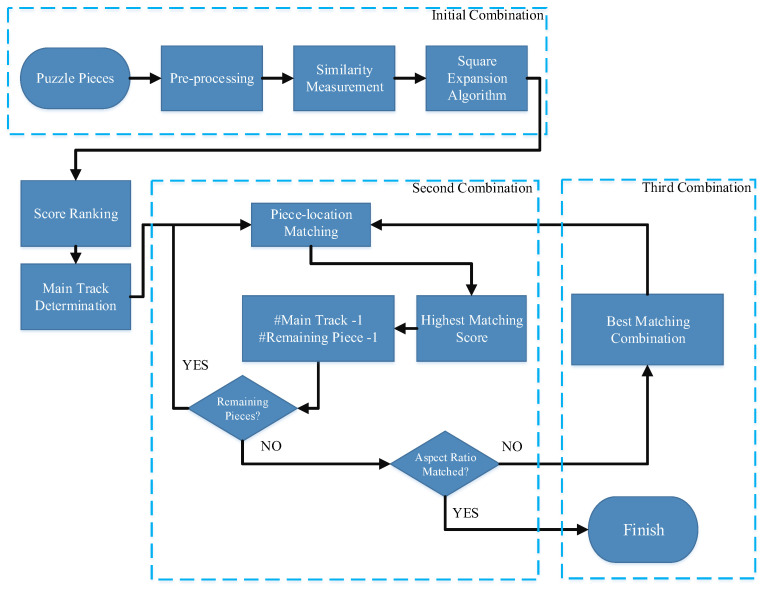
Flowchart of the proposed method without original image.

When the original image was unavailable, a feature could only be obtained by examining the color information on the edge of each puzzle piece. The flowchart of the proposed is shown in Figure 3. First, we applied the concept of the Hausdorff distance with Sobel filter similarity to select the best piece (BP) as our initial puzzle piece. Subsequently, we expanded the BP as the combination of 3 × 3 puzzle pieces with the BP as the center. Thereafter, we built an algorithm called “square expansion” to identify the initial combination. Because numerous pieces in an initial combination are usually at incorrect positions, we set a threshold to remove incorrect pieces. The remaining pieces in an initial combination were regarded as the “main track.” Subsequently, the puzzle pieces that were not in the main track were filled in the surrounding of the main track to increase the size of the main track. Finally, based on a puzzle’s size, we removed the exposed puzzle pieces and put them back to achieve local optimal results. 

The algorithm that was used when the original image was unavailable comprised four steps. The first step was searching for the BP, the second was building an initial combination and the main track, the third was refining the main track and building the second combination, and the fourth was setting the threshold for obtaining the third combination.

#### 2.2.1. Best Piece (BP) 

In the present study, the Sobel filter *H* was defined as follows:

(9) $$H=\left[\begin{array}{cc} -1 & 1\\ -2 & 2\\ -1 & 1 \end{array}\right]$$

**Figure 4:**
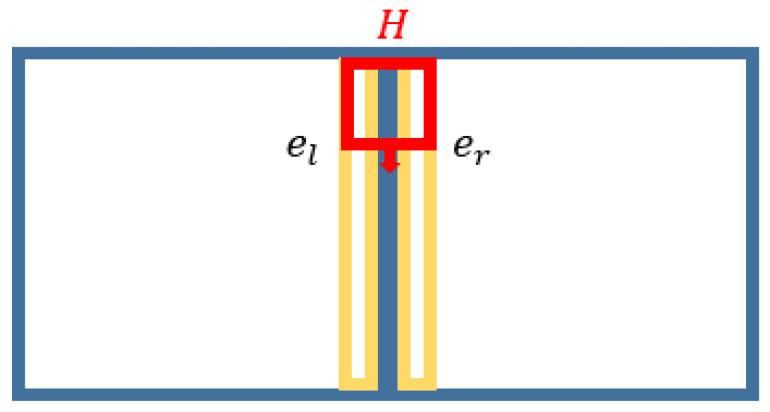
Illustration with Sobel similarity between $e_{l}^{}$ and $e_{r}^{}$, where the yellow pixels denotes the compaired pixels for filtering BP and red box denotes a slideing wondow.

We assumed that all puzzles piece were squares of length and width *n*, and $e_{l}^{}$ and $e_{r}^{}$ were the column vectors of the two adjacent edges. We merged the edges of two pieces and calculated the corresponding convolution with *H*. Figure 4 presents a diagram of the Sobel filter similarity, which is defined using the following equation:

(10) $$v_{f}=H*\left(e_{l}^{}|\;e_{r}^{}\;\right)$$

(11) $$\bar{v_{f}}=\left\|v_{f}\right\|$$

where ∗ denotes the convolution operation between *H* and $e_{l}^{}$|$e_{r}^{}$. The similarity between two edges is expressed as follows:

(12) $$Sim\left(e_{l}^{},{\;e}_{r}^{}\right)=\frac{1}{1+\bar{v_{f}}}$$

The following algorithm was based on the Hausdorff distance. However, the probability-based [27] and learning-based approaches [28] can be also taken into consideration. Instead of using the average similarity, we used the minimum value of the four edges of a puzzle piece as its score. We let *N* denote the total number of puzzle pieces. First, we randomly selected the *n*th piece as the candidate puzzle piece $P_{n}^{}$ from the total number of *N* puzzles. The four edges of the candidate piece were compared with those of other pieces. We temporarily combined the two most similar puzzle pieces via Sobel filter similarity. Thereafter, we could obtain a combination of cross-shaped pieces. The similarity of the four directions of the candidate puzzle was represented as $S_{n}^{r},S_{n}^{l},S_{n}^{u},S_{n}^{t}$ for the right, left, bottom, and top sides, respectively. The score for the *n*th piece was defined as follows:

(13) $$h_{d}\left(P_{n}^{}\right)=min(S_{n}^{r},S_{n}^{l},S_{n}^{u},S_{n}^{t})$$

The BP was expressed as follows:

(14) $$BP=\underset{n\in \left\{0,\ldots ,n\right\}}{\mathrm{argmax}}{\;\;h}_{d}\left(P_{n}^{}\right)$$

#### 2.2.2. Initial Combination

We first identified the four corners of the cross-shape corresponding to the puzzle. This step was similar to the BP search, but the edge length involved in the similarity calculation was twice that involved in the BP search. Hence, the reliability of the 3 × 3 combination was high. After the initial 3 × 3 set was completed, we built a “square expansion” algorithm, which continuously pieced a puzzle together by applying the 3 × 3 combination. We set $S_{side}\in \{S_{t},S_{r},S_{u},S_{l}\}$ as the mean similarity score for a given piece’s four directions. With the top direction serving as an example, $P_{i,j}^{t}$ is the top edge of the piece on a rectangular puzzle combination with a length and width of *m* and *n*, respectively, and $P_{i-1,j}^{u}$ is the edge of the most similar piece, as identified using $P_{m,n}^{t}$. The mean similarity score was expressed as follows:

(15) $$S_{t}=\frac{Sim\left(P_{1,1}^{t},P_{0,1}^{u}\right)+\cdots +Sim\left(P_{1,m}^{t},P_{0,m}^{u}\right)}{m}$$

(16) $$S_{r}=\frac{Sim\left(P_{1,m}^{r},P_{1,m+1}^{l}\right)+\cdots +Sim\left(P_{n,m}^{r},P_{n,m+1}^{l}\right)}{n}$$

(17) $$S_{u}=\frac{Sim\left(P_{n,1}^{u},P_{n+1,1}^{t}\right)+\cdots +Sim\left(P_{n,m}^{u},P_{n+1,m}^{t}\right)}{m}$$

(18) $$S_{l}=\frac{Sim\left(P_{1,1}^{l},P_{1,0}^{r}\right)+\cdots +Sim\left(P_{n,1}^{l},P_{n,0}^{r}\right)}{m}$$

The similarity scores in this study did not represent the final similarity value between patches. It was influenced by factors, such as the patch size and whether the patch was grayscale or colored. The average was used to find one of the most reliable edges for conducting the main track search. The range of this parameter did not necessarily span from 0 to 1, as it was used differently in this context.

**Figure 5:**
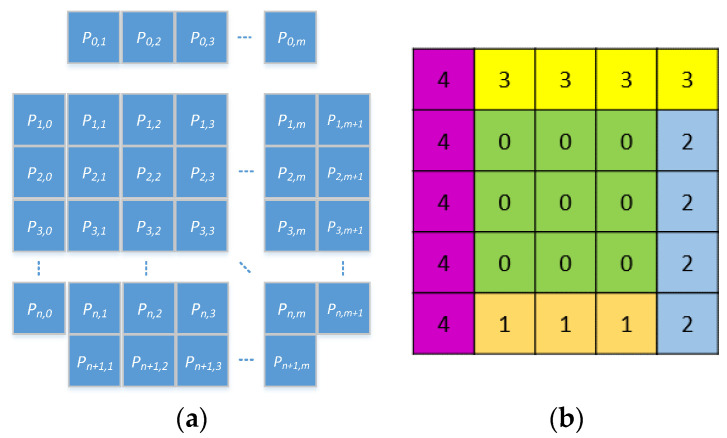
Illustration of the square expansion algorithm: (**a**) definition of each puzzle piece; (**b**) example of an update order whiche the color is only for specifying the group.

After the average similarity of the four edges was calculated, we selected the edge with the highest level of similarity with our piecing direction. For example, if ${max(S}_{t},S_{r},S_{u},S_{l})=S_{t}$, then $P_{0,0}^{\;},P_{0,1}^{\;},P_{0,2}^{\;},\cdots ,P_{0,m}^{\;}$ would be added to the top direction of the initial set. This step was repeated until all puzzle pieces were processed. Figure 5 presents an example of how the square expansion process was performed.

#### 2.2.3. Main Track

Given that the initial combination contained an excessive number of misplaced puzzle pieces and that the puzzle pieces connected to these misplaced puzzle pieces were also incorrect, we set a threshold for eliminating the puzzle pieces with a similarity level that was less than the threshold. The remaining sections of the puzzle where the misplaced puzzle pieces were removed were referred to as the main track. 

#### 2.2.4. Second Combination

After the completion of the main track, the next step was obtaining the second combination. Because the relationship between the position of the main track and the result was unknown, when the second combination was obtained, all the possible positions of the puzzle pieces were around the main track. For the second combination, the remaining puzzle pieces were placed one by one around the main track, and each puzzle piece had four possible directions. The second combination was completed when the remaining puzzles were all placed in order.

#### 2.2.5. Third Combination 

In some cases, the aspect ratio of the puzzle result was known. If the length × width of the second combination was equal to the known length × width of a rectangle, the result of the second combination was the final result. Otherwise, the rectangle was put as close as possible to the main track. The out-of-range puzzle pieces were removed and put back within the puzzle boundary. The second combination method was applied, the most similar puzzle piece was selected, and the puzzle number and rotation direction were obtained. When the remaining puzzle pieces were all placed on the puzzle, the third combination was obtained as the result.

## 3. Experimental Results

### 3.1. Experimental Setup

**Figure 6:**
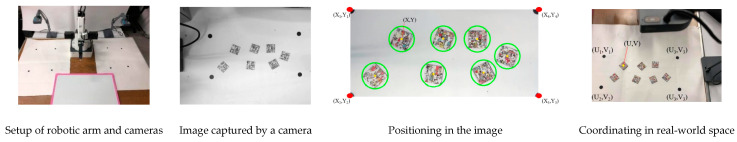
Robotic arm setup in the real world. The robot arm was placed in the middle with cameras on both sides. After we obtained the corresponding real-world position, the robotic arm placed the puzzle on the front whiteboard. The green circles denote the bounding circles of the puzzles.

We conducted all experiments using Python 3.6 and an Intel i7-4790 CPU with 10 GB of RAM. When an original image was used, we used two cameras and one robotic arm. The robotic arm was a DOBOT Arm Magician [29] with a plug-in suction cup. The setup is shown in Figure 6.

The active range of axis 4 of the robot arm was between 135° and −135°, and the initial angle $\theta_{0}$ was set to 0°. If the rotation angle was between 135° and 225°, the puzzle pieces caused an error because the end angle $\theta_{1}$ was out of range. Subsequently, the robotic arm had to put down the piece it was holding and execute another rotation. This process increased the time required to complete a puzzle. Therefore, we designed a pre-rotation table for this problem in Table 1.

We set *θ* as the rotation angle of a puzzle piece adjusted to the correct direction. If *θ* was set as 180°, the robot arm first rotated −45° to reach $\theta_{0}$. Subsequently, the suction cup of the robot arm was used to pick up the puzzle piece and to rotate its angle from −45° to 135°. Finally, *θ* (180°) was rotated without any additional rotation.

### 3.2. Result with the Original Image

We applied our puzzle assembly algorithms to a set of 10 images with different styles; they included animated pictures, natural landscapes, and jigsaw pictures with complex patterns. We first resized the images, cut them to produce multiple square puzzles, and then randomly rearranged and rotated the puzzles. Each image was formed from approximately 35 to 70 puzzle pieces, and each piece was determined to be a correct puzzle piece only if its rotation angle and placement were both correct.

SIFT feature matching uses the Euclidean distance to calculate the ratio of the closest matching feature descriptor to the second-closest matching feature descriptor. A lower threshold can obtain more feature points, whereas a higher threshold can obtain more accurate feature pairs. Therefore, we tested the images and recorded the number of correct matches of feature points under various threshold ranges. The experimental results indicated that the maximum number of successful matches was obtained when the threshold was set between 0.5 and 0.7. Although we obtained favorable results when the threshold was <0.5, several extreme cases led to highly negative results. Furthermore, we applied RANSAC to identify the correct feature pair matches and to calculate the transformation matrix. Finally, the robotic arm placed all of the puzzle pieces in what it deemed to be the correct positions.

### 3.3. Result without the Original Image

For this component of the present study, we tested the same images that were used in the previous subsection. The experimental result is presented in Table 2. First, we investigated the relationship between similarity and the initial combination. In addition, the longer the length of an edge was, the more reliable the level of similarity was. We first calculated each puzzle piece’s corresponding edge. For a 7 × 5 puzzle, 58 adjacent edge pairs needed to be calculated; for a 10 × 7 puzzle, 123 adjacent edge pairs needed to be calculated. Thus, we obtained more accurate pairs with different edge sizes and different numbers of pieces.

We also observed that larger puzzle sizes led to more accurate results. When the number of pieces increased, the accuracy ratio pertaining to the correct neighbor decreased. This occurred because numerous similar short edges resulted in the incorrect neighbor being obtained. Empirically, >50% of the correct matches could identify the BP. 

**Figure 7:**
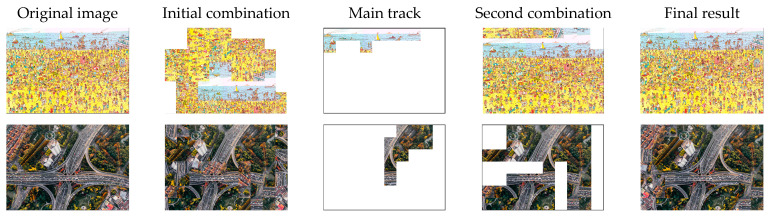
Examples of results without original images.

When we obtained an initial combination, we set a threshold to eliminate the incorrect puzzle pieces on the main track. Therefore, we conducted an experiment to determine how the threshold affected the iteration time and accuracy (Table 3). Consequently, when the threshold ratio was set to 0.9, an excessive number of pieces were filtered to form the main track, resulting in many iterations. By contrast, a low ratio led to an excessive number of incorrect puzzle pieces on the main track, resulting in unfavorable results. Our experimental results indicated that the low threshold for the 35-piece puzzle provided more favorable results because of its longer puzzle edge and its smaller number of combinations relative to the 70-piece puzzle. Thus, the main track was correct and did not require a large threshold to build a second combination. When the puzzle was smaller and the number of pieces increased, a higher threshold was required to ensure that the main track would always be correct. The step-by-step results without the original image are shown in Figure 7.

The results presented in the paper are applicable to non-rectangular puzzle pieces when the original image is available. The algorithm relies on texture features and feature point matching using RANSAC. For puzzles without an original image, non-rectangular pieces have an advantage, as additional edge shape information can be utilized in the algorithm, making it a shape-priority approach with edge pattern similarity as a supplementary matching method. Consequently, non-rectangular puzzle pieces are better suited for our foundational algorithm.

In some applications of jigsaw puzzle solving (e.g., archaeological images), the presence of noise can affect the accuracy of the feature matching and effectiveness of the feature point detection. Especially for puzzles with an original image, noise can introduce matching errors. However, for archaeological applications, where edge similarity is the primary method, noise can cause matching inaccuracies. To address this, we increase the reliance on edge shape similarity to determine whether two fragments are adjacent.

## 4. Conclusions

Solving a jigsaw puzzle is a challenging task in computer vision research, and it can be applied to the stitching or reconstruction of fragmented images. Algorithms for solving jigsaw puzzles were classified into two types, namely, those involving the use of the original image and those not involving the use of the original image. The algorithms that used the original image calculated the SIFT features on puzzle pieces and the original image and used the RANSAC algorithm to determine correct matches. The corresponding transformation matrix was calculated, and the corresponding coordinates for the robotic arm were obtained to place each of the puzzle pieces. By contrast, the algorithms that did not use original images were more difficult to use; this was because they had to solve NP-hard problems to calculate the arrangement of all puzzle pieces. We calculated the Sobel similarity between all matching edge pairs. The initial combination, the main track, the second combination, and the third combination were sequentially calculated by using a greedy algorithm to complete a puzzle. For the experimental results obtained using the original image, we performed a test to rebuild a jigsaw puzzle using a robot arm in real-world settings, and we achieved excellent results for most of the analyzed images. For the experimental results obtained without the use of original images, we tested the effect of various thresholds for speed and accuracy and achieved more favorable results relative to those of other studies. The proposed algorithm exhibited versatility in handling puzzle pieces of various shapes, including those with high color variance, complex textures, and even grayscale images. Its potential applications extend to industrial settings, particularly in tasks like visual inspection and mechanical assembly, where the assistance of robotic arms can be effectively utilized.

## Figures and Tables

**Table 1:** Pre-rotation table.

Range of θ	$\theta_{0}$ (Start Angle)	$\theta_{1}$ (End Angle)
135° ~180°	135°–θ	135°
− 135° ~135°	0 (as default)	θ
− 180°~− 135°	− 135°–θ	− 135°

**Table 2:** Accuracy of adjacent pieces combined during the initial combination.

#Pieces (#Neighbors)	Method	Puzzle Size (Pixel)	#Correct Neighbors	#Wrong Neighbors
35 (58)	Ours	>100	53	5
		60~100	33	25
	Cho et al. [15]	>100	17	41
		60~100	18	40
70 (123)	Ours	60~100	76	47
		<60	73	50
	Cho et al. [15]	60~100	28	95
		<60	26	97

**Table 3:** Mean iteration counts and puzzle completion accuracy with diferent threshold ratios.

	Method	Threshold Ratio	0.1	0.2	0.3	0.4	0.5	0.6	0.7	0.8	0.9
35 pcs	Ours	Iteration	7	19	23	29	30	32	33	33	34
		Puzzle completion accuracy	87.1%	80%	80%	69.1%	69.1%	69.1%	76.8%	76.8%	80%
	Cho et al. [15]	Iteration	19	22	27	27	28	31	33	35	37
		Puzzle completion accuracy	11.1%	11.1%	21.7%	53.3%	66.3%	74.6%	86%	77.8%	77.8%
70 pcs	Ours	Iteration	12	20	23	28	29	32	32	33	35
		Puzzle completion accuracy	61.9%	64.4%	77.8%	50.1%	62.5%	62.5%	62.5%	50	62.5%
	Cho et al. [15]	Iteration	27	45	56	56	61	62	65	68	71
		Puzzle completion accuracy	12.6%	12.5%	12.6%	46.3%	62.5%	62.5%	73.6%	73.6%	71.6%

## Data Availability

Not applicable.
